# Determination of Acceptor Concentration, Depletion Width, Donor Level Movement and Sensitivity Factor of ZnO on Diamond Heterojunction under UV Illumination

**DOI:** 10.1371/journal.pone.0089348

**Published:** 2014-02-26

**Authors:** Kim Guan Saw, Sau Siong Tneh, Fong Kwong Yam, Sha Shiong Ng, Zainuriah Hassan

**Affiliations:** Nano-optoelectronics Research Laboratory, Universiti Sains Malaysia, Minden, Penang, Malaysia; Gazi University, Turkey

## Abstract

The concentration of acceptor carriers, depletion width, magnitude of donor level movement as well as the sensitivity factor are determined from the UV response of a heterojunction consisting of ZnO on type IIb diamond. From the comparison of the I-V measurements in dark condition and under UV illumination we show that the acceptor concentration (∼10^17^ cm^−3^) can be estimated from p-n junction properties. The depletion width of the heterojunction is calculated and is shown to extend farther into the ZnO region in dark condition. Under UV illumination, the depletion width shrinks but penetrates both materials equally. The ultraviolet illumination causes the donor level to move closer to the conduction band by about 50 meV suggesting that band bending is reduced to allow more electrons to flow from the intrinsically n-type ZnO. The sensitivity factor of the device calculated from the change of threshold voltages, the ratio of dark and photocurrents and identity factor is consistent with experimental data.

## Introduction

In recent years there has been considerable interest in ZnO-based UV detectors. Previous efforts include the fabrication of Schottky photodiode using (0001) single crystal ZnO and epitaxial thin film as well as p-n homojunctions involving mono-doping with group V elements such as Sb, N, P, and As [1]–[5]. The advantages of ZnO-based UV detectors are due to the attractive properties of ZnO such as a wide band gap (∼3.3 eV), high exciton binding energy (∼60 meV) and a relatively non-toxic nature. However, one of the challenges of using ZnO-based devices is the intrinsic n-type conduction of ZnO due to the effect of self-compensation. As-deposited ZnO shows n-type conductivity due to excess electrons from defects usually attributed to Zn interstitials, O vacancies, hydrogen or hydrocarbons. Acceptor carriers in ZnO can thus be easily compensated. In addition, low solubility of dopants and the deep levels introduced by the acceptors usually cause a low carrier concentration. Other problems in fabricating p-type ZnO are associated with the so-called aging effect [6]–[8] where the ZnO material subsequently reverts to n-type conductivity.

One of the potential p-type materials for ZnO-based heterojunction devices is diamond. It is chemically stable and does not form an interfacial layer with ZnO, which could affect device performance. ZnO and diamond have wide band gaps of 3.37 and 5.47 eV, respectively. P-type conductivity in diamond is usually caused by boron impurities that exist naturally as in the case of natural type IIb diamond or introduced into the diamond by the process of doping during chemical vapor deposition. Boron seems to be a suitable acceptor and with sufficient B a narrow impurity band is formed. The activation energy of B is ∼0.37 eV. At low boron concentrations, reasonable carrier activation can be obtained for doping level of 10^13^ cm^−3^ [9]. In principle, a concentration of 1 ppm of B or an estimated carrier concentration of 10^17^ cm^−3^ has been known to have a semiconducting nature and is able to cause p-type conductivity [10]. Type IIb diamond is known to be semiconducting. Thus ZnO can be combined with diamond to make a p-n heterojunction using their intrinsic properties with the added advantage of diamond as a stable material with a high thermal conductivity of ∼22 W cm^−1^K^−1^. Despite this interesting possibility, the electrical behavior of a heterostructure consisting of ZnO and diamond seems elusive. Hikavyy *et al*. [11] reported a rectifying behavior only for very lightly doped p-type chemical vapor deposition (CVD) diamond using residual boron gas for doping during the chemical vapor deposition process but intentional (higher) doping with B did not result in a heterojunction displaying a rectifying behaviour. Unfortunately, the acceptor concentrations in both types of samples were not determined. Other studies reported a rectifying behaviour for heterojunctions with p-type CVD diamond where carrier densities are between 10^18^–10^19^ cm^−3^ [12]–[13]. Very recently Huang et al. [14] reported that while such heterojunctions displayed a rectifying behaviour, they were not sensitive to visible or UV illumination under forward conditions. No significant changes were observed for the threshold voltage in dark condition as well as UV illumination. In this study we investigate the UV response of a heterojunction consisting of as-sputtered ZnO on type IIb diamond where no intentional doping is involved. The intrinsic donors in ZnO and acceptors in the type IIb diamond are thus involved in the conduction of current. This relatively simple configuration enables us to determine the device properties such as the concentration of the acceptor carriers, depletion width, donor level movement and sensitivity factor.

## Materials and Methods

The diamond substrates used in this work were type IIb (100) crystals measuring 2.5 mm×2.5 mm×0.5 mm. The as-received diamond substrates were cleaned using ethanol, acetone as well as deionized water sequentially in an ultrasonic bath and dried using pure nitrogen gas. The surface of the diamond substrate was partially sputtered with a thin film of ZnO of approximately 500 nm in thickness using a pure ZnO target in a pure argon atmosphere. Current-voltage (I–V) measurements were performed in the dark and under UV illumination at room temperature using the Keithley I-V measurement system. The intensity of the UV illumination provided by a UV lamp (λ = 372 nm) was approximately 5 mW cm^−2^. X-ray photoelectron spectra were recorded using monochromatized Al Kα (1486.7 eV) x-ray radiation while micro-Raman spectroscopy and x-ray diffraction measurements were done using the Renishaw RN 1000 model and the PANalytical X'pert PRO high resolution x-ray diffractometer system respectively. An argon ion line at 514.5 nm was used as the excitation source for Raman measurements while the XRD measurements were performed with a fixed copper anode operating at 40 kV and 30 mA. The X-ray diffraction data was collected using Cu Kα radiation.

Initial characterizations of the sputtered ZnO thin films, metal contacts, diamond quality and evidence of boron in the type IIb diamond have been reported in our previous work [15]. The x-ray excited auger electron B KLL peak at the kinetic energy range of 176–184 eV is within the range observed in previous studies [16]. Raman measurements shows a peak at 1332.3 cm^−1^ with a full width half maximum of 4.5 cm^−1^, confirming that the diamond is of good quality. Since the Raman peak is Lorentzian symmetric, a low concentration of boron of about 10^17^ cm^−3^ can be inferred. The boron concentration is thus well below the threshold of ∼2×10^20^ cm^−3^, the so-called Mott density, which corresponds to the onset of metallic conductivity [9]–[10]. A concentration above the threshold value will result in an asymmetric peak shape or the so-called Fano-like lineshape which is caused by a quantum mechanical interference between the zone-centre Raman active optical phonon and the continuum of electronic states induced by the dopant [17].

## Results and Discussion

**Figure 1:**
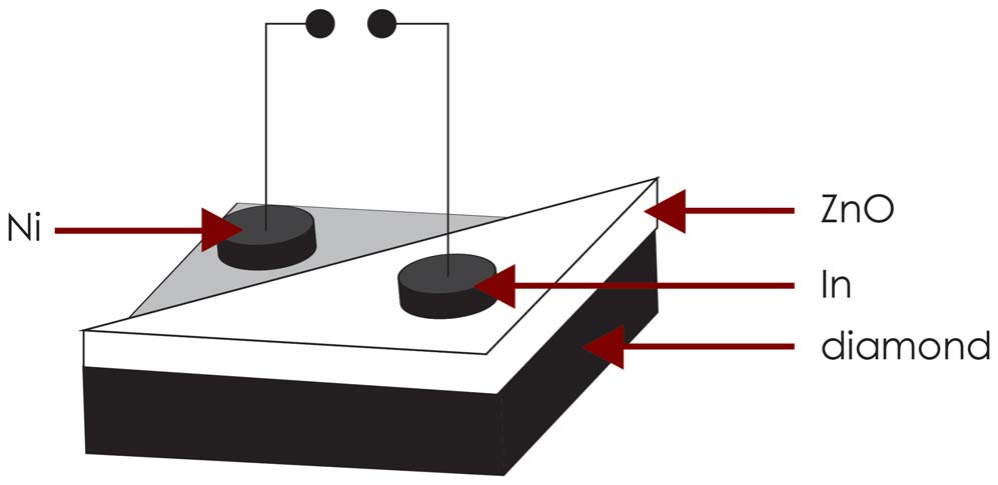
Schematic diagram of ZnO/type IIb diamond heterojunction.

The schematic diagram of the ZnO on type IIb diamond heterojunction is shown in Fig. 1.

XRD measurements show the ZnO (002) and the diamond (111) peaks at 2θ∼34.331° and 44.576° respectively. The peak positions are in good agreement with those in the JCPDS card file data for hexagonal wurtzite ZnO (No. 36-1451) and cubic diamond (No. 6-0675). A continuous film of ZnO has been formed on the type IIb diamond as evident from the SEM analysis. No contaminants were detected in the ZnO thin film or diamond substrate using EDS analysis. I - V measurements of the In and Ni metal contacts indicate that ohmic contacts are established on the ZnO thin film and type IIb diamond, respectively, after annealing the contacts in air in a controlled furnace at 600°C for 3 min.

**Figure 2:**
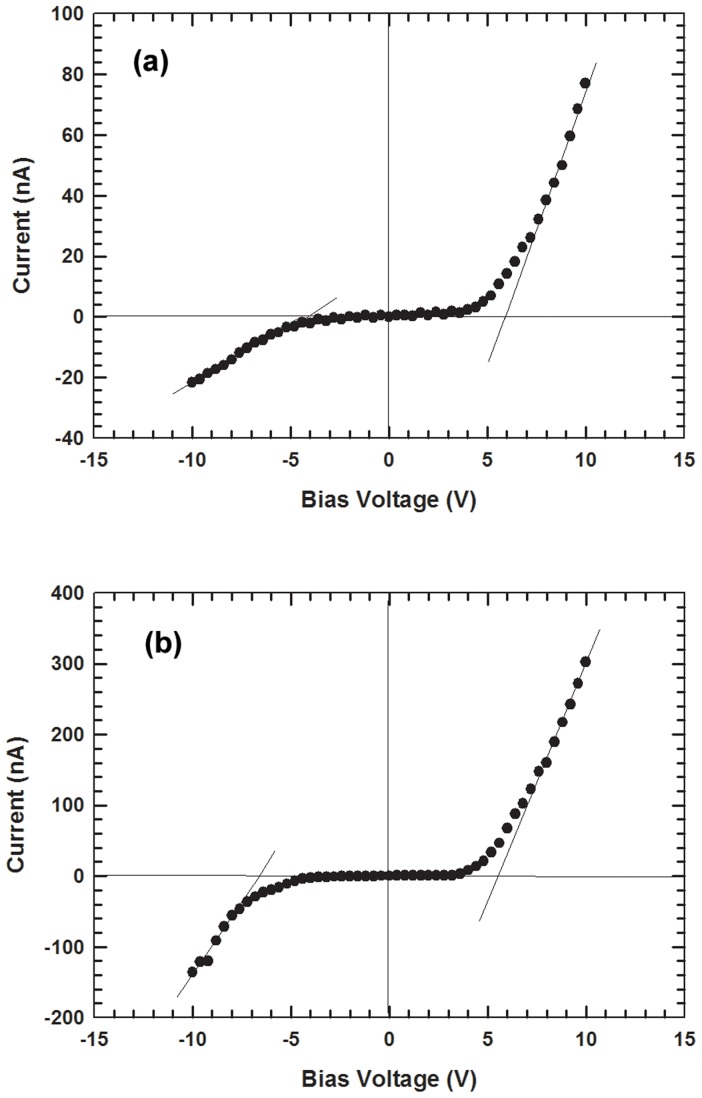
I–V behavior of the ZnO on type IIb diamond heterojunction under (a) dark condition and (b) UV illumination.

**Figure 3:**
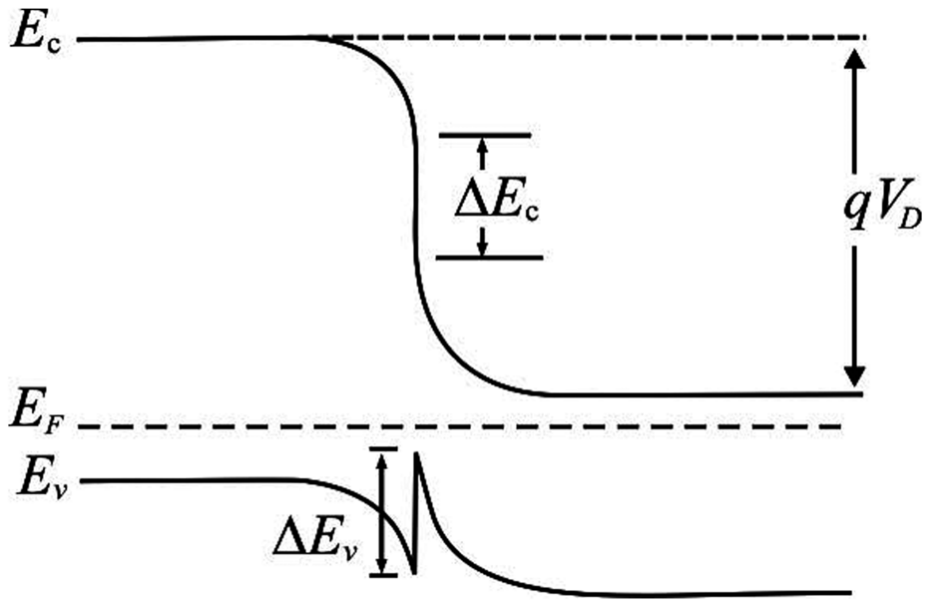
Energy band diagram of the ZnO on type IIb diamond heterojunction.

The I – V measurements of the ZnO on type IIb diamond heterojunction show that forward conduction begins when the applied voltage reaches ∼4.0 V for both dark and UV conditions. The extrapolated threshold voltages under dark and UV conditions are 5.9 and 5.5 V, respectively while the reverse breakdown voltages are −4.6 and −6.6 V, respectively (Fig. 2(a) and (b)). The relatively high threshold voltage is believed to be due to high series resistance and diffusion voltage. This outcome is expected since both materials are not heavily doped or intentionally doped. At the reverse voltage of 10V, the current for UV condition is 135 nA while only 22 nA is observed for dark condition. An insight of the electrical behavior can be obtained by studying the band diagram of both the materials that form the heterojunction. Fig. 3 shows the band diagram of the ZnO on type IIb diamond heterojunction. Since the band gap of diamond is larger than that of ZnO, the bending of the conduction band is significantly bigger than the bending of the valence band.

The conduction band offset is
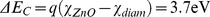
(1)while the valence band offset is
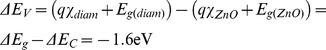
(2)The negative value indicates a discontinuity in the joining of the valence bands.

It should be noted that for lower doping densities in diamond, holes are thermally excited from’ acceptor states into the valence band with an activation energy of 0.37 eV. Here conduction occurs via holes in the valence band contributed by the ionized B. Based on the band gap diagram, under forward current, the energy spike and potential well impede the movement of holes and the forward current thus increases slowly with the applied voltage. In the reverse bias, as the applied voltage increases the bottom of the valence band of diamond approaches the bottom of the valence band of ZnO and thus the energy spike and potential well becomes narrower, enabling the holes to tunnel through them easily. Thus a relatively large reverse current is observed.

**Figure 4:**
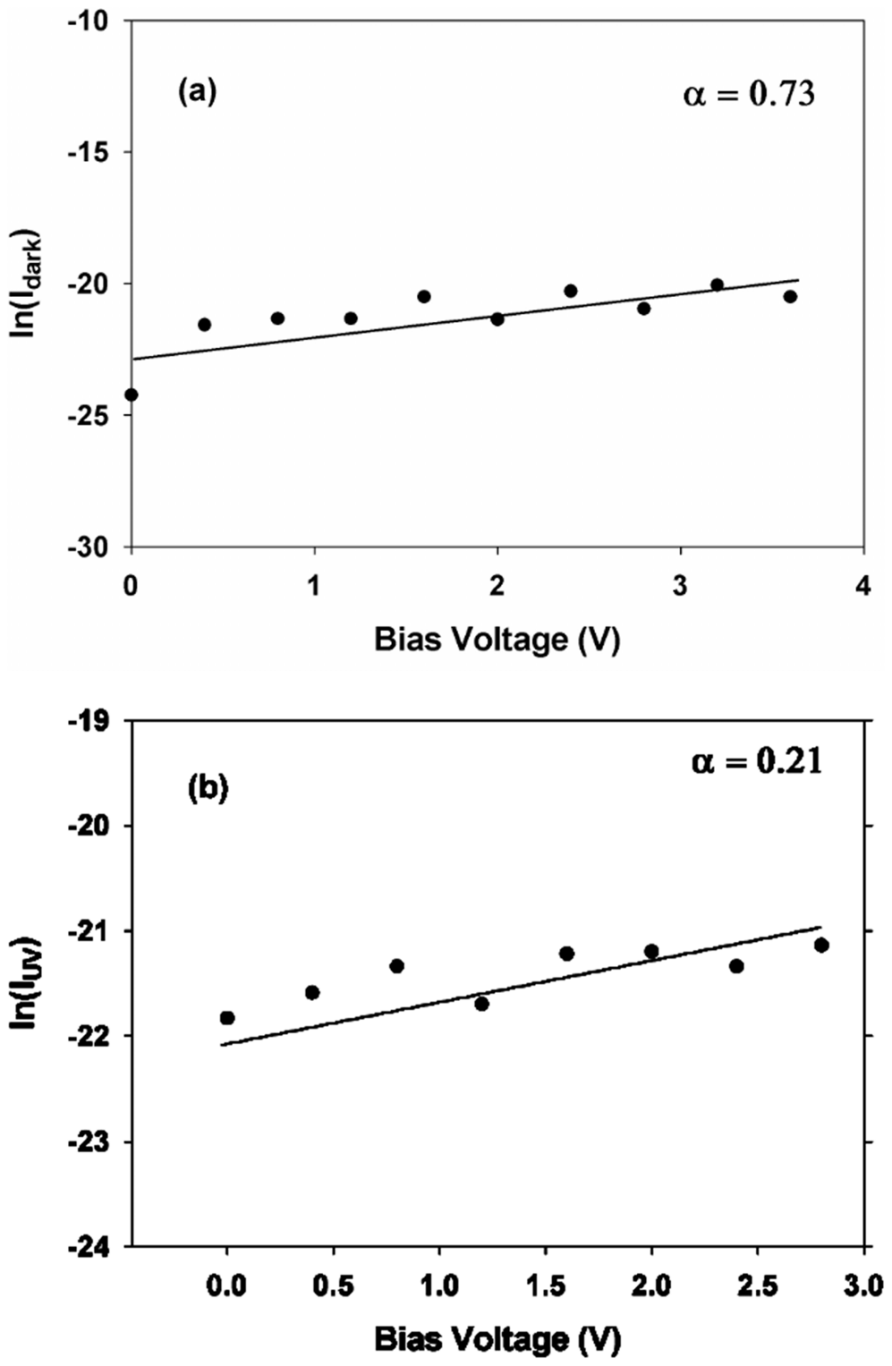
The determination of α for (a) dark condition and (b) UV condition.



The increased current flow of an order higher under UV illumination is expected. The current increases expontentially as 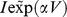
where α is a constant [18] and is given as
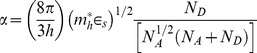
(3)where 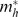 is the effective mass of holes, 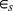
is the dielectric constant of ZnO, 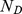
is the donor concentration, 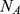
the concentration of acceptors and *h* is the Planck constant. Since the diamond material is not significantly involved during UV illumination because its large band gap is greater than the energy of the UV light (3.33 eV) the concentration of acceptors
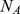
is regarded as constant and it can be estimated from the values of α obtained in dark condition as well as under UV illumination. Assuming that 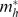 and 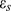
are constant under both these conditions we write
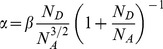
(4)where 
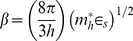
(5)Under the condition 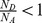
we obtain
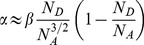
(6)Using 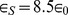 [19] and from curve fitting, α is found to be 0.73 and 0.21 V^−1^ for dark condition and under UV illumination, respectively (Fig. 4(a) and (b)). Assuming 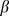 remains the same for dark condition as well as under UV illumination, we obtain

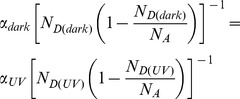
(7)


The donor concentration
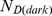
for dark condition obtained from Hall Effect measurements is 3.93×10^16^ cm^−3^ while the concentration under UV illumination 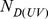 is 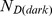
plus the number of donors contributed by the volume of ZnO under UV illumination that result from the breaking of the electron-hole pairs. The latter can be estimated from the photocurrent measurements. Since the dimensions of the ZnO thin film are known, its volume under illumination can be easily obtained, which is ∼1.17×10^−7^ cm^3^. (Note that the ZnO material under the In contact is excluded).

The photocurrent that is caused by the UV illumination alone when the applied voltage is 0 V is
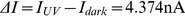
(8)By assuming that one photon creates only one electron-hole pair during UV illumination in ZnO and considering 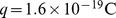, the additional number of electrons due to the UV illumination is 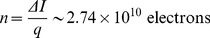
(9)The additional carrier concentration of donors (electrons) is 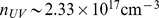 and thus the total donor concentration under UV illumination is 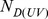
is 2.72×10^17^ cm^−3^. Substituting these values we obtain
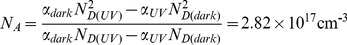
(10)The calculated value of 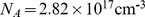
is reasonable for a semiconducting type IIb diamond. Note that the condition 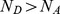
will not have a physical meaning as it gives a negative value of 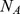.

The UV illumination also causes changes in the depletion or transition region W. In the depletion region some electrons diffuse from the ZnO material into diamond while some are swept by the electric field from the diamond to ZnO material. Since the electric field sweeps out carriers that have wandered into the transition region, the space charge in this region are the uncompensated donor and acceptor ions. To fulfil the equal charge requirement, the depletion region thus extends into the diamond and ZnO regions unequally.

For electrostatic cases the depletion widths inside diamond and ZnO can be expressed respectively as [20]

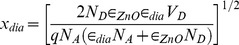
(11)and
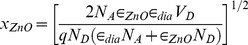
(12)where *q* is the charge of the electron, *V_D_* is the potential and 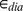 and 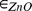
are the dielectric constants of the diamond and ZnO materials. The total depletion width (zero bias) for the heterojunction is simply the addition of both these regional depletion widths, which is
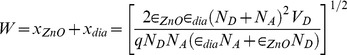
(13)In dark conditions, the ratio of the penetration depths is
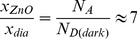
(14)This suggests that the depletion region extends farther into the ZnO region, which is the side with the lighter doping. Under UV illumination, the penetration ratio approaches unity implying that the depletion region penetrates both materials equally. Here
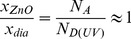
(15)Using 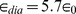 [21] the depletion width is found to be 348 nm. However, under UV illumination, the depletion width is observed to have shrunk to 162 nm. It can be shown that the shrinking of the depletion width in the ZnO/type IIb diamond heterojunction is due to enhanced carrier density as a result of UV illumination. Oxygen is adsorbed on the surface of the ZnO particles which have a relatively high surface-to-volume ratio and traps or captures the excess electrons present in the intrinsic n-type semiconducting material, forming negatively charged ions according to the equation
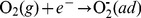
(16)Note that 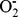 and not 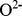 is formed. It should be noted that the adsorption of oxygen molecules on the oxide surface has also been previously suggested as a trapping mechanism involved in the enhancement of carrier density and photoconduction in ZnO nanostructures [22]–[23]. The process of oxygen adsorption and subsequent formation of negatively charged ions 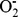 also causes band bending upwards near the surface. Upon illumination with a photon energy that is larger than the ZnO bandgap, electron-hole pairs are produced. 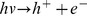
(17)These photogenerated holes migrate to the surface along the potential slope caused by band bending and discharge the negatively charged adsorbed oxygen ions according to the equation
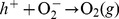
(18)


The photogenerated holes are thus captured by the negatively charged adsorbed oxygen ions. The unpaired electrons left behind thus have the same density as the captured holes. The discharge of the negatively charged oxygen ions causes the photodesorption of oxygen from the surface. The carrier density is increased due to a large amount of photogenerated electrons. We believe that the increased density of carriers subsequently decreases the depletion width of the ZnO/type IIb diamond heterojunction. Since the applied voltage determines the amount of charge stored in the junction and the number of charge carriers per cm^−3^ has increased, less width is needed to store the required charge in the junction.

The donor level movement can be obtained from the diffusion voltage which is related to the threshold voltage 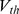, current *I* as well as the series resistance 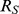
as
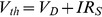
(19)where 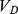
is the diffusion voltage and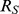 is the series resistance. From the energy band diagram, 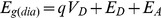
(20)where 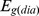 is the energy band gap of diamond, 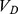
is the diffusion voltage while 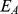 and 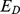 are the boron acceptor level above the valence band in diamond and donor level in ZnO below the conduction band, respectively. Taking the values of 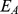 and 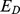 as 0.37 and 0.38 eV respectively [12] we obtain 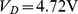
for dark condition. The diffusion voltage is within the expected value as forward conduction occurs when the applied voltage reaches ∼4.0 V and the extrapolated threshold voltage occurring at 5.9 V for dark condition. With the assumption that the diamond material is not involved during UV exposure, Equation (20) can be simplified as
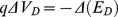
(21)Note that 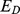
refers to the donor level from the bottom of the conduction band. In ZnO, the concentration of donors 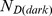
is related to the quasi Fermi level 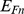 as 
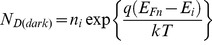
(22)




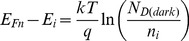
(23)Here 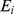
is the intrinsic Fermi level, 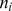 the intrinsic carrier concentration, *q* is the charge of an electron, *k* the Boltzmann constant and *T* the absolute temperature. Under UV illumination, the electron-hole pairs are generated in ZnO thin film and the electrons subsequently affect the quasi-Fermi energy of the ZnO. Thus
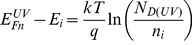
(24)The change in the quasi Fermi level is thus
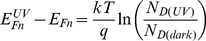
(25)Note that 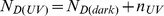 where 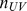
is the additional number of donors that result directly from the breaking of electron-hole pairs during UV illumination. Thus
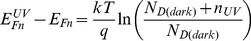
(26)The donor level from the bottom of the conduction band is related to the quasi Fermi level as 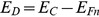
(27)The difference in the quasi Fermi levels before and after UV exposure is related to 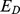
as
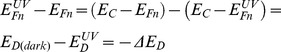
(28)Thus the change of the donor energy level 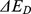
in *n*-ZnO under UV illumination can be expressed as
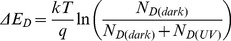
(29)Subsequently we obtain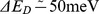. The negative sign implies that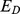 has moved closer to the conduction band under UV illumination. The difference in the voltage threshold values between dark condition and under UV illumination from experimental results is ∼0.4 V.

The sensitivity factor, *S*, can be found from the ratio of the dark and photocurrents during forward bias. Noting that
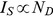
(30)and 
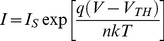
(31)The ratio of the dark and photocurrents is
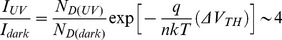
(32)where 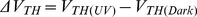


**Figure 5:**
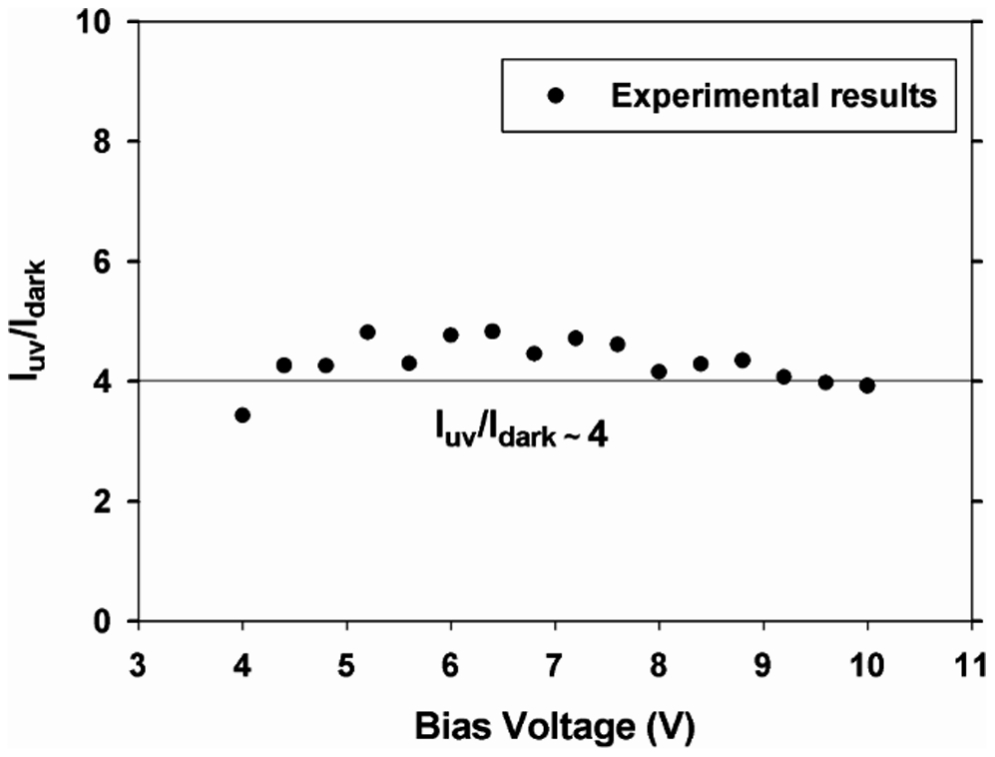
The ratio of I_UV_/I_dark_ obtained from current measurements.



Fig. 5 shows the ratio of the currents which is determined from experimental data is approximately 4. The theoretical consideration above is therefore substantiated with evidence from current measurements. The sensitivity factor is thus

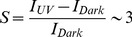
(33)


## Summary

We have successfully investigated the UV response of a heterojunction consisting of ZnO and type IIb diamond and demonstrated that the p-n junction properties such as concentration of acceptor carriers, depletion width and sensitivity factor can be determined from I – V measurements. The UV illumination changes the concentration of donors and subsequently enables the acceptor concentration to be calculated. The depletion width decreases by about 50%. Band bending is possibly reduced resulting in the donor level moving closer to the conduction band by 50 meV. The ratio of I_UV_/I_dark_ obtained from calculations collaborates with experimental data leading to a sensitivity factor of approximately 3.
